# Advocating for Lesbian, Gay, Bisexual, and Transgender Youth Sexual and Reproductive Health and Rights in Central Asia

**DOI:** 10.9745/GHSP-D-24-00207

**Published:** 2024-12-20

**Authors:** Ulukbek Batyrgaliev

**Affiliations:** a Eurasian Coalition for Health, Rights, Gender and Sexual Diversity, Tallinn, Estonia.; b KDI School of Public Policy and Management, Sejong, Republic of Korea.

## Abstract

Lesbian, gay, bisexual, and transgender youth in Central Asia face challenges due to the current sociopolitical context, and there is a pressing need for legal and policy reforms to align with the International Conference on Population and Development agenda.

## Key Messages

- Central Asian sociopolitical setting presents challenges in providing sexual and reproductive health services that cater to the needs of lesbian, gay, bisexual, and transgender (LGBT) youth.
- Existing legal frameworks and societal norms in Central Asia contribute to discrimination against LGBT youth, resulting in limited access to health care services.
- Advocacy efforts and international support play a role in driving inclusive policy reforms aimed at enhancing health outcomes for LGBT youth.

## INTRODUCTION

The International Conference on Population and Development (ICPD) held in 1994 set forth an approach to sexual and reproductive health and rights (SRHR) that focuses on incorporating rights into global health policies. In Central Asia, lesbian, gay, bisexual, and transgender (LGBT) youth (aged 15–24 years) encounter resistance from political players in having their sexual and reproductive health (SRH) needs met. This resistance is purposely reflected in public discourse, underscoring the disparity between the ICPD objectives and the SRHR context in the region.^1^ Despite advancements in legislation addressing SRHR for the general population—such as measures to reduce maternal and infant mortality rates and increase access to modern contraceptive methods—many Central Asian countries still lack specific laws prohibiting discrimination based on sexual orientation and gender identity. This gap is influenced by the region’s prevailing social norms and legal landscape, which often neglects the unique needs of LGBT communities.^2^ Access to services, such as social assistance, education, and mental health support, is hampered by conservative beliefs that ignore or discriminate against gender identities and sexual orientations. In Kazakhstan, Kyrgyzstan, and Tajikistan, rooted societal heteronormative norms pose challenges to embracing and providing LGBT health care services, often leading to discrimination and marginalization.^3^ In Uzbekistan and Turkmenistan, LGBT relationships are criminalized, which fosters an environment of fear and hinders LGBT individuals’ access to health care services.^4^^–^^6^ The absence of disaggregated data on gender and sexual diversity, as well as lack of population estimates of men who have sex with men^7^ or LGBT individuals, further exacerbates the challenges and reinforces assumptions and misconceptions.^8^ These issues affect LGBT youth the most because of their economically vulnerable position in society and higher susceptibility to abuse and discrimination.

A shortage of competent LGBT SRH providers is another obstacle that the region’s health care system faces. This deficit, in turn, impacts the quality and availability of services provided to LGBT youth, resulting in elevated risks of sexually transmitted infections and other health issues. A report by the Eurasian Coalition on Health, Rights, Gender, and Sexual Diversity (ECOM), an international nongovernmental membership association supporting LGBT human rights, health, and social well-being in Eastern Europe and Central Asia, highlighted vulnerabilities faced by young transgender individuals in accessing HIV services. The report stated that they often encounter additional layers of discrimination and transphobia both within health care settings and in the LGBT community, which complicates their ability to receive appropriate care.^8^ In these cases, community groups and grassroots movements are essential in offering support, education, and advocacy for LGBT rights. When public health systems fall short, these groups often step in to drive and advocate for policy changes at the country level. To address disparities effectively on a global scale, international organizations, like the World Health Organization and the Joint United Nations Programme on HIV/AIDS, are incorporating specific policies and health indicators tailored to LGBT populations into their evaluations. These measures aim to identify gaps in access to care, reduce stigma in health care settings, and ensure that health services are equitable and inclusive.^9^^,^^10^ These actions encourage governments to tackle discrimination and marginalization of the LGBT population in their countries and emphasize the pressing need for initiatives and legal reforms to promote a more accepting and supportive environment.

In this article, I draw on my background as a regional SRHR expert, sexual orientation, gender identity, and expression and human rights advocate, as well as on my experience working with organizations focused on SRHR for LGBT youth in Central Asia. Through collaborations with international organizations and my position as SRHR Coordinator at ECOM, I have gained insights into the sociopolitical and legal landscapes that shape access to health and rights in these contexts. For each country in Central Asia, I provide an overview of the current regime and examine its legal framework regarding human rights, LGBT rights, and SRHR. Finally, I suggest ways to improve the issues through the implementation of the ICPD mechanisms.

## THE INTERNATIONAL CONFERENCE ON POPULATION AND DEVELOPMENT AND LESBIAN, GAY, BISEXUAL, AND TRANSGENDER YOUTH

The ICPD brought forth a Programme of Action that reshaped population policies by putting rights at the forefront of demographic goals.^11^ This shift emphasizes how the principles of the ICPD apply to issues impacting LGBT youth. Although the Programme of Action did not directly address LGBT rights, its strong focus on ensuring freedoms and ending discrimination laid a foundation for advocating for the rights of LGBT youth today.^11^ These principles support SRH education, indirectly aiding initiatives for LGBT youth by promoting an understanding of diversity and respect for individual rights. Such educational efforts play a crucial role in creating environments that protect LGBT individuals from discrimination and violence while ensuring they have access to health services.^11^ Implementing these principles today involves advocating for policies that acknowledge and cater to the needs of LGBT youth within health care and education programs, which aligns with the ICPD’s position against discrimination and for the promotion of individual rights. The ICPD Programme of Action underscores the relevance of its approach in addressing the struggles faced by LGBT youth and championing rights and safeguards within the context of human rights and progress.^11^

Although the ICPD Programme of Action did not directly address LGBT rights, its strong focus on ensuring freedoms and ending discrimination laid a foundation for advocating for the rights of LGBT youth today.

## CENTRAL ASIA COUNTRY ANALYSIS

### Kazakhstan

Kazakhstan continues to operate under a semi-authoritarian regime despite some surface-level reforms that suggest a move toward democratization. The political system is mainly controlled by the ruling elite, and elections are often criticized for lack of competition and accusations of manipulation.^12^ Civil liberties, such as freedom of the press and the right to gather, are tightly restricted, making it challenging for any significant opposition movements to emerge. The health care system has made progress toward accessibility through greater funding, but considerable barriers persist, particularly in rural areas where sexual health education frequently fails to reach international norms.^13^

The environment for LGBT youth in Kazakhstan poses challenges to their health and security. Legal protections are inadequate, and societal acceptance remains low. United Nations experts have expressed concern over a petition in Kazakhstan that calls for banning LGBT “propaganda,” warning that such a law would severely infringe on human rights, including freedoms of expression and assembly.^14^ The petition highlights deeper issues within the country’s semi-authoritarian regime, where civil liberties are already tightly restricted, and political reforms, while visible, often lack substantive democratization.

This restrictive environment complicates the situation for LGBT youth, who face significant challenges in accessing health care and social services due to widespread discrimination and legal inadequacies.^14^ Even though the Kazakhstani Constitution outlines principles of equality and nondiscrimination, they are not effectively put into practice, especially concerning LGBT rights and HIV issues.^8^ The country’s legal framework does not offer protection against discrimination based on sexual orientation and gender identity. Discrimination and harassment are widespread in schools and public spaces, making it difficult for LGBT youth to access social services.^15^ As a result, LGBT individuals encounter challenges in obtaining health care, which violates Kazakhstan’s human rights commitments and poses a health risk by restricting essential services for vulnerable populations.^16^ Although these services are included in the state’s free health care offerings, the lack of an antidiscrimination law creates significant gaps in health care access.^17^ Positive efforts made by the government, like implementing an action plan on rights in December 2023, show progress toward establishing an antidiscrimination task force. However, there is a need for greater legal reforms, realignment of public health policies, and changes in societal attitudes to ensure that LGBT youth in Kazakhstan can lead dignified lives and have equal access to all societal benefits, including health care.^17^

### Kyrgyzstan

Although Kyrgyzstan was once considered a beacon of democracy in Central Asia, the current government’s commitment to human rights has regressed. Recent securitized political changes, oppression of the media, nongovernmental organizations that receive foreign funding, and the elimination of opposition in the government have resulted in a consolidation of authoritarian regimes in the country.^18^^–^^21^ The decline in democratic governance and human rights protection is evident through the increased government influence over the media and public life, limiting opportunities for participation and individual freedoms.^22^ Most importantly, LGBT youth encounter societal prejudice and legal obstacles. The surge in conservative rhetoric has put the rights and safety of LGBT individuals at risk with direct and structural discrimination and barriers to their well-being and fundamental rights. The country lacks laws to protect against discrimination based on sexual orientation and gender identity, making LGBT individuals vulnerable to various forms of mistreatment and violations of their rights. Instances of hostility toward them are common in public and private spaces, schools, and homes, leading to their exclusion and mental health struggles.^23^ The lack of safeguards is worsened by proposed legislation—“the traditional values” and another one commonly known as “foreign agents”—that could further limit the rights of LGBT individuals and economic dignity to live a desired life.^24^^–^^27^ Additionally, societal attitudes toward LGBT individuals in Kyrgyzstan tend to be negative due to norms that disapprove of traditional sexual orientations and gender identities. These biases are often evident in the media and public discussions, leading to discrimination and sometimes sparking violence against LGBT individuals. The health care system in Kyrgyzstan mirrors these views, with LGBT individuals encountering obstacles when trying to access health care. The issues related to SRHR in Kyrgyzstan are characterized by disparities between urban and rural areas, lack of comprehensive policies, and problems with the availability of health care services and addressing the health care needs of young people and women.^28^ There are instances where health care providers have refused service solely based on a person’s sexual orientation or HIV status, which not only violates basic human rights but also hinders access to essential health care services for LGBT individuals. Discrimination continues without recourse for those affected due to the perpetuation of preexisting prejudices in this environment.^29^ Therefore, there is an urgent need to align international organizations’ attention to LGBT youth’s SRHR in Kyrgyzstan.

### Tajikistan

Tajikistan’s government, led by President Emomali Rahmon, has sustained an authoritarian political environment devoid of opposition, independent media, and public discourse on criticism of the government.^22^ Despite LGBT relationships being decriminalized in 1998, discrimination and social stigma persist due to gaps in legislation and insufficient protective measures for issues related to people’s sexual orientation and gender identity. Further, the government’s intelligence agency compiled lists of people with assumed sexual orientation and exposed it publicly, endangering LGBT people to mistreatment, physical violence, and death threats.^30^ The National Report on Violations of LGBT People’s Rights and MSM in Tajikistan provides instances where legislative frameworks poorly prevent discrimination against LGBT youth activists who are subject to detention under Article 125 of the Criminal Code.^31^

The situation regarding SRHR in Tajikistan is complex due to rooted traditional norms and minimal government involvement in crucial areas like family planning and sexual education. This leads to other challenges, including high rates of adolescent pregnancies and limited availability of contraceptive methods, which, in turn, impacts the well-being and financial autonomy of women and young people.^32^ While there are some safeguards for individuals living with HIV under the Health Code, mandatory HIV testing, in the context of employment and education restrictions, is discriminatory.^31^ The 2022 law against discrimination does not explicitly mention sexual orientation and gender identity as part of the protected categories. This leads to uncertainty and a lack of enforcement in safeguarding the rights of LGBT individuals. International society should pressure Tajikistan’s government to recognize all sexual orientations and gender identities as protected individuals under antidiscrimination law, decriminalize HIV transmission according to global norms, and provide better training for law enforcement officers and health care professionals on LGBT matters.^33^

### Turkmenistan

Turkmenistan is known for being one of the most closed-off and authoritarian countries in the world. The government tightly controls all aspects of society, from the media to political parties and civil organizations, leaving no room for dissent or opposition.^22^ The situation for LGBT individuals in Turkmenistan is harsh because laws and societal norms criminalize and stigmatize homosexuality. LGBT youth live in fear of being exposed and persecuted without any legal protection or support systems.^34^ The stigma against LGBT individuals also extends to health care settings, where they often face discrimination from health care workers, making it difficult for them to access health services tailored to their needs.^34^ Discussions on SRHR are almost nonexistent due to government oversight of health care information and services. This discrimination discourages many from seeking care in areas related to health and mental well-being. A study conducted by a nongovernmental organization, Kyrgyz Indigo, reported that the restrictive legal environment is worsened by established biases; LGBT individuals often encounter familial rejection and exclusion from communal engagements and become frequent victims of violence.^5^ The absence of safeguards or avenues for redress significantly amplifies their susceptibility and seclusion, highlighting a necessity for systemic reforms to safeguard and assist LGBT individuals in Turkmenistan. It is crucial for health care providers to embrace practices to enhance the accessibility of health services for the LGBT community.

### Uzbekistan

Despite some signs of progress toward openness in the government, Uzbekistan still operates under a strict authoritarian system. Changes in politics are slow. The government usually cracks down on dissenting media outlets and censors human rights agenda presentations to their benefit.^22^ Men in Uzbekistan who engage in consensual same-sex sexual conduct are subject to arbitrary detention, prosecution, and imprisonment under Article 120 of the criminal code. This discriminatory legal framework exacerbates the challenges faced by the LGBT community. The current laws provide no protections against discrimination based on sexual orientation or gender identity. Additionally, the criminalization of same-sex relations between men compels LGBT youth to hide their identities and navigate a rigidly heteronormative and binary-gendered society.^4^ Incidents from 2023 include violations of LGBT rights, with state officials or civilians perpetrating cases of harassment and discrimination on physical and psychological levels. These violations are rarely addressed effectively, undermining trust between the LGBT community and government bodies. Many young people in the LGBT community face detention, physical harm, and coercion often carried out by law enforcement officers.^35^ This leads to an array of mental health issues, such as increased levels of anxiety, depression, and other mental disorders, affecting LGBT individuals.^4^ SRHR issues, particularly past practices like forced sterilization, are being addressed; however, full access to health care services remains hindered by coercion and discrimination.^10^ It is imperative to create communities and safe spaces to improve the well-being and safety of LGBT individuals by providing them with social and emotional support.^4^ Most importantly, there is a call for changes in laws and policies to offer protection to LGBT people. This involves revising laws that harm the LGBT community, implementing discrimination policies, and promoting public acceptance and understanding.

## CONCLUSION

Reflecting on the 3 decades since the ICPD in Cairo, Central Asian LGBT youth still face significant barriers to accessing SRHR due to restrictive legal systems, societal discrimination, stigma, and violence. The country-specific LGBT-related challenges highlighted in this article reveal a pressing need for targeted engagement in the region. These conservative social ideals often disregard LGBT communities, criminalize their existence, and restrict access to health care and fundamental rights.

The country-specific LGBT-related challenges highlighted in this article reveal a pressing need for targeted engagement in the region.

Coinciding with the 30th anniversary of the ICPD, in 2024, events like the ICPD Global Youth Dialogue and the Summit of the Future served as key platforms for advancing discussions on SRHR.^36^^,^^37^ With its theme, “a new generation’s vision for the future,” the Global Youth Dialogue gathered young advocates, policymakers, and community leaders from diverse backgrounds to share their insights, identify ongoing challenges, and advocate for inclusive, youth-centered policies. This Dialogue amplified youth voices and highlighted the specific needs of marginalized communities, aiming to shape the future of SRHR policies and create supportive, inclusive environments. My attendance at the Global Youth Dialogue, as well as at the United Nations Economic Commission for Europe Regional Reviews for the 25th and 30th anniversaries of ICPD and the High-Level Commission on the Nairobi summit on ICPD25, has provided me with firsthand insights into the impact of these platforms, strengthening the foundation for the recommendations presented in this article.

Although this article primarily focuses on HIV-related issues, which are highly prevalent and disproportionately affect LGBT communities, there is comparatively less attention to other sexual health needs, such as sexually transmitted infections. This limited focus is partly due to the lack of comprehensive data on the SRHR needs and challenges of LGBT individuals in Central Asia, as well as the fact that HIV-related programs receive more support and funding thanks to Global Fund and U.S. President’s Emergency Relief Fund for AIDS Relief. Addressing HIV alone is insufficient; a comprehensive SRHR approach must include accessible and equitable services across all areas of sexual health, which remain largely unavailable for LGBT individuals in the region.

Regionally, there are additional issues regarding LGBT individuals’ access to different forms of contraception, hormone therapy for transition, human papillomavirus vaccinations, and in vitro fertilization treatments. Regrettably, these individuals face significant stigma, leading to a lack of data collection and lack of research on being stigmatized or monitored by the state, forcing the LGBT community to endure ongoing challenges, experience higher mortality rates, and perpetuate the cycle of poverty in the region. The global community, including activists and policymakers, needs to increase their efforts to break down the prevalent cultural, legal, and political obstacles hindering the fulfillment of SRHR for LGBT youth. Embracing awareness and rights-focused strategies will play a pivotal role in advancing toward a more inclusive and fairer SRHR landscape in Central Asia.

To bridge the gaps in ICPD’s SRHR agenda, Central Asian states should require inclusive health care policies, guaranteeing LGBT youth catered services that honor their identities and needs. It is imperative for the community of legal professionals and human rights advocates to prioritize aligning SRHR services with broader human rights agendas and turn the attention of future ICPD-related initiatives toward promoting LGBT health and rights. LGBT youth are in the most vulnerable crust of the society. Effective approaches to advocating for them may involve using human rights platforms to influence actions, increasing support for local nongovernmental organizations, and cultivating regional partnerships to champion inclusive policies. The promotion of the visibility and acceptance of LGBT identities through education and public health campaigns will vastly enhance the health and well-being of LGBT youth in the area, advancing the general well-being and development in the region.

## Notes

**Peer Reviewed**

**First Published Online:** November 29, 2024.
